# Systems Medicine: from molecular features and models to the clinic in COPD

**DOI:** 10.1186/1479-5876-12-S2-S4

**Published:** 2014-11-28

**Authors:** David Gomez-Cabrero, Jörg Menche, Isaac Cano, Imad Abugessaisa, Mercedes Huertas-Migueláñez, Akos Tenyi, Igor Marin de Mas, Narsis A Kiani, Francesco Marabita, Francesco Falciani, Kelly Burrowes, Dieter Maier, Peter Wagner, Vitaly Selivanov, Marta Cascante, Josep Roca, Albert-László Barabási, Jesper Tegnér

**Affiliations:** 1 Unit of computational Medicine, Center for Molecular Medicine, Department of Medicine, Karolinska Institute and Karolinska University Hospital, Stockholm, Sweden; 2 Center for Complex Network Research, Northeastern University Physics Department, Boston, MA 02115, USA; 3 Department of Theoretical Physics, Budapest University of Technology and Economics, H-1111 Budafoki út. 8., Budapest, Hungary; 4 Hospital Clinic, IDIBAPS, CIBERES, Universitat de Barcelona, Barcelona, Catalunya, Spain; 5 Barcelona Digital Technology Centre Carrer Roc Boronat, 117 08018 Barcelona; 6 Institut d'Investigacions Biomediques August Pi i Sunyer (IDIBAPS), Barcelona, Spain; 7 Institute of Integrative Biology, University of Liverpool, Crown Street, Liverpool, UK; 8 Department of Computer Science, University of Oxford, Wolfson Building, Parks Road, Oxford, OX1 3QD, UK; 9 Biomax Informatics AG, Munich, Germany; 10 School of Medicine, University of California, San Diego, San Diego, CA 92093-0623A, USA; 11 Center for Cancer Systems Biology, Dana-Farber Cancer Institute, Smith Bldg., Rm. 858A, 450 Brookline Ave, Boston, MA 02215, USA; 12 Center for Network Science, Central European University, Nadoru. 9, 1051 Budapest, Hungary; 13 Channing Division of Network Medicine, Brigham and Women's Hospital, Harvard Medical School, 181 Longwood Avenue, Boston, MA 02115 USA

**Keywords:** Chronic diseases, COPD, Disease heterogeneity, Systems Medicine, Predictive Modeling, Co-morbidity

## Abstract

**Background and hypothesis:**

*Chronic Obstructive Pulmonary Disease *(COPD) patients are characterized by heterogeneous clinical manifestations and patterns of disease progression. Two major factors that can be used to identify COPD subtypes are muscle dysfunction/wasting and co-morbidity patterns. We hypothesized that COPD heterogeneity is in part the result of complex interactions between several genes and pathways. We explored the possibility of using a Systems Medicine approach to identify such pathways, as well as to generate predictive computational models that may be used in clinic practice.

**Objective and method:**

Our overarching goal is to generate clinically applicable predictive models that characterize COPD heterogeneity through a Systems Medicine approach. To this end we have developed a general framework, consisting of three steps/objectives: (1) feature identification, (2) model generation and statistical validation, and (3) application and validation of the predictive models in the clinical scenario. We used muscle dysfunction and co-morbidity as test cases for this framework.

**Results:**

In the study of muscle wasting we identified relevant features (genes) by a network analysis and generated predictive models that integrate mechanistic and probabilistic models. This allowed us to characterize muscle wasting as a general de-regulation of pathway interactions. In the co-morbidity analysis we identified relevant features (genes/pathways) by the integration of gene-disease and disease-disease associations. We further present a detailed characterization of co-morbidities in COPD patients that was implemented into a predictive model. In both use cases we were able to achieve predictive modeling but we also identified several key challenges, the most pressing being the validation and implementation into actual clinical practice.

**Conclusions:**

The results confirm the potential of the Systems Medicine approach to study complex diseases and generate clinically relevant predictive models. Our study also highlights important obstacles and bottlenecks for such approaches (e.g. data availability and normalization of frameworks among others) and suggests specific proposals to overcome them.

## Introduction

Recent years have seen a paradigm shift in Life Sciences: *"from a fragmented to a systems approach, linear to nonlinear methodology and from genome to physiome based analysis" *[1]. Systems Medicine, as an adaptation and extension of *Systems Biology*, embraces this paradigm and is becoming a cornerstone in the study of complex diseases. A general introduction to Systems Medicine is provided in [2]. In this article, part of a Supplement dedicated to the Synergy-COPD project [2], we review and assess the Synergy-COPD's Systems Medicine approach to study Chronic Obstructive Pulmonary Disease (COPD), both a chronic and a complex disease.

While the characterization of COPD has been extensively investigated and there is a continuous refinement of guidelines (e.g. GOLD), there is yet no consensus on a phenotypic definition of the term "COPD patient". For instance in [3] several sub-types of COPD patients were identified; see also [4] within this Supplement for further details on the heterogeneity in COPD. Briefly, within Synergy-COPD we aim to characterize two sources of heterogeneity: first we investigated the systemic effects associated with skeletal muscle dysfunction in COPD patients (*MusclDYS*). Second, we aimed to characterize co-morbidity patterns of COPD patients (*CoMorb*). Finally, we also investigated the interplay between the different heterogeneities, which may provide a novel description of COPD as being driven by the interaction between several factors.

Using COPD as a case-study we explore the notion that Systems Medicine provides tools to investigate and characterize disease heterogeneity. To this end our analyses follow a general three-step procedure: (1) first, we need to identify the relevant biomarkers (or more generally *features of interest, FoI*) for each case of heterogeneity; (2) in a second step, predictive models with the potential to be applied in the clinic are developed and validated statistically. Third and final, (3) the usefulness of the models in a clinical scenario has to be validated. To achieve this goal we integrated a wide variety of available resources, such as prior domain knowledge, relevant data-bases and existing probabilistic and mechanistic models.

The rest of this article is organized as follows: the next section details the Systems Medicine framework used in Synergy-COPD. The third and fourth sections describe the application of this framework in the characterization of *MusclDYS *and *CoMorb*. These sections include a brief description of the questions, the obtained results and the limitations of the proposed methodology. The final section provides the conclusions and summarizes the identified remaining challenges.

## The Synergy-COPD's systems medicine approach

**Figure 1:**
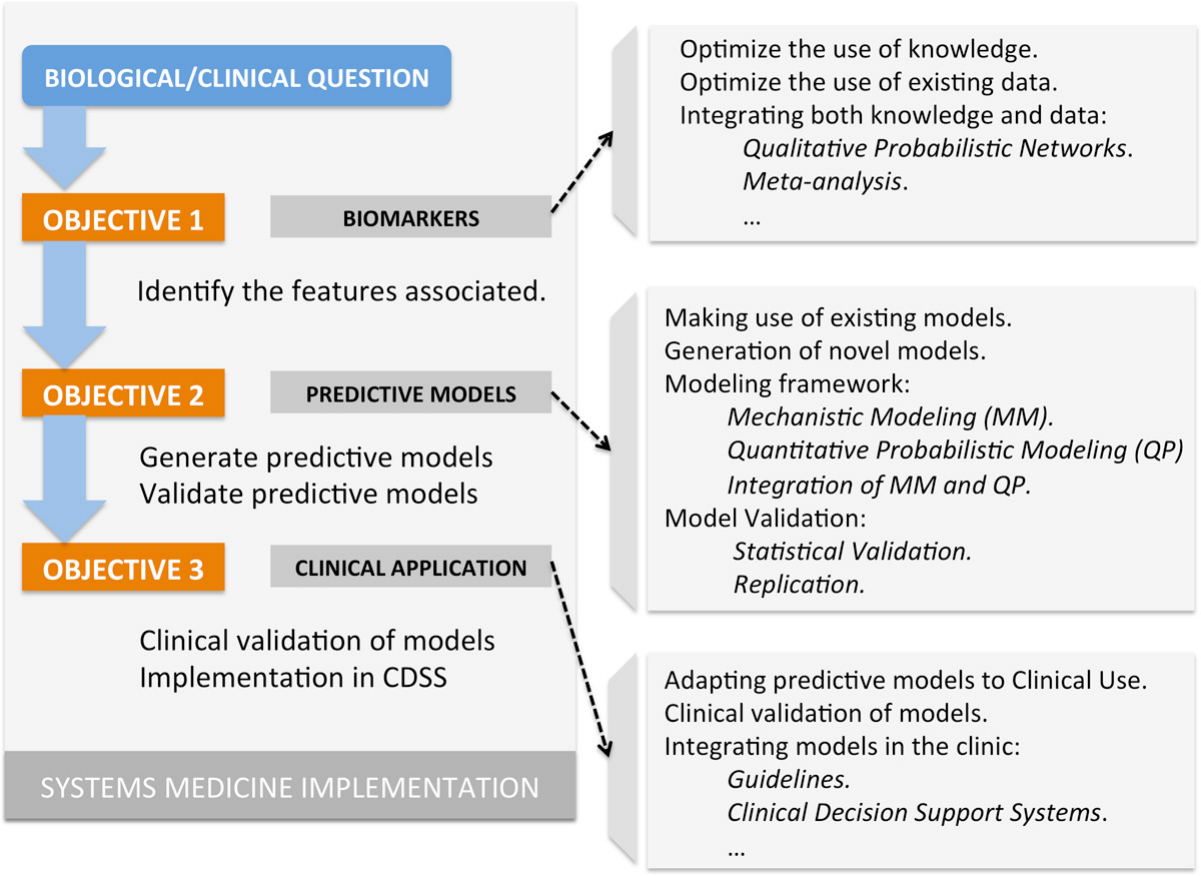
**Synergy-COPD's Systems Medicine framework**.

Systems Medicine provides a comprehensive and general framework to investigate the complex interactions implicated in human disease in an integrated fashion. Consequently, there is no single defined set of methodologies associated with Systems Medicine. Instead, any methodology useful to investigate the question under study "as a system" can be considered as relevant to explore and validate. While the concrete focus of Synergy-COPD lay on COPD, we aimed at developing a more general framework that may also be applicable to other complex diseases. We therefore started by defining a generic three-step objective plan that sets the goals in our studies of *MusclDys *and *CoMorb *(Figure 1). The plan was then concretized and adapted to each question accordingly.

Our final goal was a characterization of the disease heterogeneities that can be transferred to clinical practice (Figure 1, Objective 3), in particular by implementing it into a Clinical Decision Support system (CDSS, [5]). The first step (Objective 1) towards this goal is to identify the relevant, *i.e*. most predictive, biological features among the large amount of available data, *e.g. *genes, metabolites and clinical variables, among many others. The second step (Objective 2) is to integrate these features into predictive models and to validate them. In the following we briefly review each objective for the two case studies *MusclDys *and *CoMorb *and introduce the respective different resources and methodologies that were used.

**Objective 1**, (Biomarker identification): having defined a question of interest (e.g. *MusclDys*) we first need to identify the relevant associated features. The core of this Objective is formed by publicly available data-sets and knowledge (e.g. Gene Omnibus [6]) that were integrated into a user-friendly knowledge-base [7]. The different methodologies for *MusclDys *and *CoMorb *are detailed in two separate sections.

**Objective 2 (Predictive modeling)**: the identified features are now used in predictive models that may provide insights into the question of interest. For instance, in *MusclDys *we aim to predict the effects of muscle dysfunction in a given patient. In *CoMorb *we aim to compute the probability of developing a specific co-morbidity in COPD patients. Those quantitative models are question-specific and require both statistical (*e.g. *through cross-validation [8]) and biological validation.

**Objective 3 (Clinical application)**: bridging the gap between a predictive model and its use in clinical practice constitutes an important and challenging task: Beyond the basic statistical and biological validation of a model, it also needs to be clinically relevant in the context of personalized medicine. In this objective, predictive models are reviewed for their possible uses in a CDSS. Once a model is considered useful in principle, both a thorough clinical validation and an optimal CDSS implementation are required.

## Understanding COPD skeletal muscle dysfunction (*MusclDys*) through systems medicine

### Objective 1: Biomarker identification

To identify the relevant features associated with muscle dysfunction/wasting we used existing data and knowledge through (existing and novel) network-based methodologies. We considered the Biobridge clinical study [2] as the core of the data and extended it through publicly available data-sets from GEO [6]. Among the most relevant data-sets are the gene expression profiling of sputum in COPD ex-smokers (http://www.ncbi.nlm.nih.gov/geo/query/acc.cgi?acc=GSE22148) and Peripheral Blood Mononuclear Cell (PBMC) profiling of COPD patients by COPDgene [9]. For those data-sets used but not publicly available we had permission to access and analyze the data. Next we describe the interactome-based methodologies and results.

#### The Interactome

**Figure 2:**
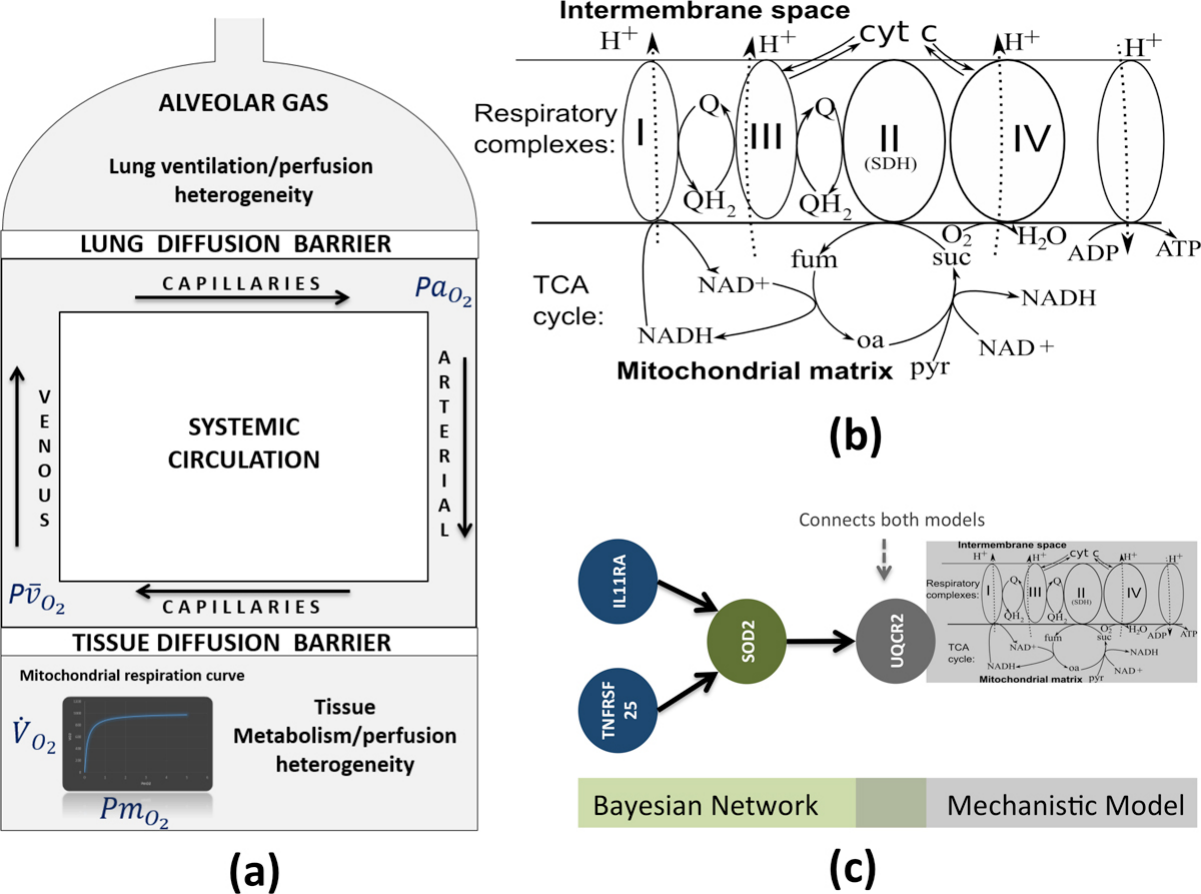
**Mechanistic Models and extensions**. (a) Oxygen transport and utilization model (M1). (b) Mitochondrial respiration and reactive-oxygen species generation model (M2). (3) Personalized model of M2: M2 model is personalized by a Bayesian network that predicts the values of UQCR2 (oxidative phosphorylation chain) by inflammation-associated measurements (IL11RA and TNFRSF25). All models can be simulated in the Simulation Environment [58] and the patient specific values can be obtained through a COPD Knowledge Based [7].

The etiology of COPD involves a multitude of intertwined molecular processes, many of which still remain unknown. These processes are embedded in the larger context of the *Interactome*, referring to a single comprehensive network integrating all molecular interactions, such as protein-protein interactions, regulatory protein-DNA interactions or metabolic interactions (see Figure 2). While ongoing efforts to systematically map the complete Interactome stand only at the beginning, currently available databases (Table 1) already include several hundreds of thousands of interactions. In order to explore such large networks, Systems Medicine has extensively adopted tools from network science [10-12]. Network approaches to human disease are based on the observation that the cellular components associated with a specific disease are not scattered randomly within the Interactome, but segregate in certain neighborhoods or *disease modules*. The identification of the specific disease modules is therefore an important step towards a holistic understanding of how molecular variations with small isolated effect sizes collectively give rise to a certain disease phenotype. The local agglomeration of disease-associated proteins within the Interactome can be used in this process, by extrapolating from the connectivity patterns of known disease-associated proteins to infer novel disease proteins [13,14]. Other applications of this principle include the identification of pathway members [15] or prioritization of weak GWAS loci [16]. In [17] a COPD specific protein interaction network was constructed around genes differentially expressed between healthy and COPD subjects. This network was then queried for potential drug targets that could reverse the expression changes. COPD specific gene expression is also the basis of a Systems Medicine method proposed in [18] that aims at identifying subgroups of COPD patients with different molecular signatures.

**Figure 3:**
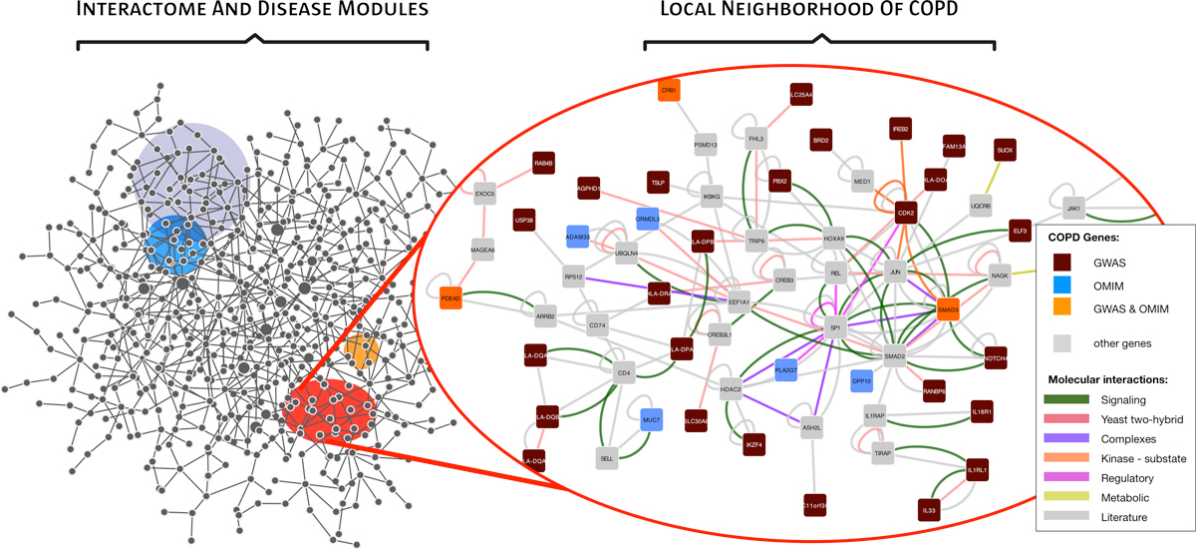
**The "Disease Interactome"**. The Interactome (left) represents the complex network of all molecular components (gene products, proteins, metabolites, RNAs etc.) and their interactions. Diseases can be understood as local perturbations. The local neighborhood around genes reported to be associated with COPD (right) is only a very small subset of the full Interactome, yet already shows its enormous complexity.

**Table 1:** Major resources for protein interaction data:

Database / Interaction type	Reference
Databases integrating several sources	IntAct [59], MINT [60], BioGRID [61], HPRD [62], MIPS [63], STRING [64]
Protein complexes:	CORUM [65], [66]
Binary interactions (high-throughput)	CCSB-HI (CCSB), [67;68]
Regulatory interactions	TRANSFAC [69]
Kinase-substrate Interactions	PhosphositePlus [70]

Typically, only direct *physical *(binding) interactions are considered in the Interactome. Another line of Systems Medicine network approaches uses *functional *networks, where links may also represent indirect associations, for example co-expression [19] or genetic interaction [20,21]. These networks are usually assembled from specific experimental data, rather than the more general interaction data in public databases. An example for the use of functional networks in COPD is given by the study of [22]. In order to explore the molecular basis of muscle degeneration in COPD, they developed a network model integrating several types of relevant measurements, such as blood cytokine levels and muscle gene expression. They were thereby able to identify several tissue remodeling and bioenergetics pathways that fail to coordinate in COPD diseased muscles.

Both physical and functional-based networks are useful tools that allow the identification of de-regulated functional elements, and to zoom-in into the interactions driving that de-regulation. We considered this information to be relevant in the generation of predictive models addressing the characterization of heterogeneity in COPD.

#### Methodologies and results

To characterize skeletal muscle dysfunction in COPD patients before and after training we evaluated two hypotheses. In the first hypothesis, *MusclDys *is characterized by the de-regulated activity of a selected set of pathways; namely *transcription, proteolysis, immune activation *and/or *oxidative phosphorylation *(see [4] for more details). Using these pathways as a reference an initial list of associated genes was downloaded from the Synergy-COPD Knowledge-base SKB [7] and then filtered by clinical and biological experts. We followed a network approach similar to one described in the BioBridge analysis [22] and extended it by including differential expressed genes, metabolites, cytokines, and clinical variables in addition to the filtered list of genes. We generated a network for each combination of healthy *vs *COPD and untrained *vs *trained individuals, obtaining two main results. First, we identified a module (*i.e*. a network-based cluster of genes, *Mod*1) that is present in all networks and shows the interaction between *mRNA-translation, Insulin *and *mTOR *pathways. Interestingly, this module was also associated to immune markers such as *IL1B*. A second result is the general loss of co-regulation (including *Mod *1 loss) in COPD patients' muscle after training, independently confirming [22]. These promising results require further corroborating data in order to obtain a robust predictive model.

In a second hypothesis, we considered that given the relevance of bioenergetics and immune markers in COPD patients, we could use them to explain the level of *Reactive Oxidative Species *(ROS, major markers of oxidative stress) in COPD patients' muscle. In order to identify the sub-network(s) linking immune and bioenergetics genes to ROS-associated genes (from [23;24]) in the context of COPD we developed a chain-based methodology [25] named ChainRank. Briefly, the methodology identifies relevant sub-networks by identifying and scoring chains of interactions that link specific targets. The type of interactions to include in the chain search are selected by the user; we selected among those interactome-networks included in the Synergy-COPD Knowledge-Base [7]. Scores are generated from the integration of multiple general and context specific measures. Finally, the algorithm allows the identification of genes that are over-represented in highly ranked chains as relevant features. This list was then used for generating personalized predictive models in Objective 3.

### Objective 2: Predictive models

The generation of predictive models in *MusclDys *involves three steps, beginning with the identification of existing mechanistic models that were then updated and adapted to better estimate particular features of interest (FoI). In this process we make use of Objective 1's results to either identify FoI or to personalize the models by adding disease-related parameters.

#### The identification of mechanistic existing models

Many phenomena in physiology and biology are of essentially nonlinear nature and therefore require quantitative descriptions in addition to qualitative ones [26]. We considered three well-described physiological models of interest to the characterization of COPD: (i) *Oxygen transport and utilization *[27,28] (*M-OX*); (ii) *Cell Bioenergetics, mitochondrial respiration and reactive-oxygen-species generation (ROS) *[23,24], (*M-ROS*); and, (iii) *Spatial heterogeneities of lung ventilation and perfusion *[29] (*M-HET*). The first two models (*M-OX *and *M-ROS*) are relevant for the characterization of the systemic effects of the disease in skeletal muscle, as they provide mechanistic description of both the oxygen pathway and ROS generation. The third model (*M-HET*) is relevant for the study of pulmonary events in a sub-set of COPD patients [3] with low pulmonary density (high emphysema score) and mild airway remodeling resulting in mild to moderate airflow limitation.

*Oxygen transport and utilization *[27,28]*(M-OS): *The model details the determinants of oxygen transport from air to mitochondria and characterizes oxygen utilization at mitochondrial level during maximum exercise. In summary, it constitutes the most complete integrative approach of the interplay among factors modulating oxygen transport (lungs, hearth, blood and skeletal muscle) and oxygen utilization at muscle level (see Figure 2(a)). In COPD patients, the oxygen transfer capacity from the atmosphere to the cell, as well as its utilization at mitochondrial level, can be limited. It is therefore of interest to observe the effects of such a limitation at all levels of the transport chain [30] and, in particular, its effects on muscle's mitochondria.

*Cell Bioenergetics, mitochondrial respiration and reactive-oxygen-species generation (M-ROS): *The model details mitochondrial respiration and its relation with the production of Reactive Oxidative Species (ROS). The model integrates two sub-models, the Electron Chain model and the TCA cycle module [23,24]. ROS production in the mitochondrial respiratory chain is a signal of cellular adaptation to the environment, but a sharp increase is incompatible with cell survival; therefore the predicting ROS production is a relevant task. Moreover, in smoking-related COPD patients the antioxidant capacity is severely reduced and further decreases after smoking cessation due to endogenous production of ROS [31,32]. The integrated model (M-OX + M-ROS) generated within the Synergy-COPD project allows estimating quantitatively the relationships between determinants of cell oxygenation and mitochondrial ROS generation. The interplay between ROS levels and the antioxidant capacity of the redox system ultimately determines tissue oxidative and nitrosative stress with important implications on pathway regulation and cell damage.

*Spatial heterogeneities of lung ventilation and perfusion *[29] (M-HET): The anatomy-based multi-scale model of the human pulmonary circulation allows for the study of pre- and post-occlusion flow and embolus-generated blow flow redistribution, among other features. It combines four independent simulations of model geometry, tissue mechanics, ventilation and blood flow allowing for a local description of alveolar ventilation and pulmonary blood flow. The lung modeling approaches are described in this Supplement in detail in a separate paper as interactive work with the FP7 EU Project *AirPROM*. A major relevance of lung modeling in Synergy-COPD is the characterization of patients with reduced lung density but without classical COPD symptoms of airway obstruction [3]. The inclusion of this modeling approach in Synergy-COPD had two main goals. Firstly, the analysis of the impact of spatial heterogeneities of lung ventilation and perfusion on blood oxygenation and, secondly, the study of the subset of COPD patients showing dissociation between high emphysema score and low intensity of airway remodeling, as indicated above and described in [3].

#### Updating existing models

In order to characterize skeletal muscle dysfunction in COPD (*MusclDys*) we modified the oxygen transport and utilization model (M-OX) and included the mitochondrial respiration [33]. The outcomes of this model were two fold: (1) it increased the physiological validity of the model by estimating the mitochondrial P_O2_, (2) it allowed for the integration with bioenergetics models (M-ROS). A second extension of the model was the modeling of lung ventilation/perfusion heterogeneities and [33]; these extensions (see Figure 2(a)) allows better estimation and better personalization of the model in COPD.

In addition, we integrated models M-OX and M-ROS (IM) to model the relation between oxygen transport and ROS generation, which constitutes a major marker of skeletal muscle dysfunction. Parameter models were investigated again in the case of [23,24], to provide better ROS estimations. A major outcome of the integrated model analysis [34] is that it permits to estimate the effect of various states of oxygen supply and demand of mitochondrial P_O2 _on ROS production; this is relevant in COPD patients with airway obstruction symptoms [22].

#### Novel models

In Synergy-COPD we generated novel models (Objective 2) by integrating existing mechanistic models, developed for the healthy individual, with features of interest (FoI) associated to *MusclDys *(obtained in Objective 1). Having identified ROS as a major marker of muscle dysfunction, we aimed to predict ROS status by surrogate variables identified in Objective 1. Initially those surrogate variables were immune markers from protein measurements in the blood as shown in [22]. To integrate the effect of the surrogate variables (SV) with the integrated model of oxygen transport and ROS generation we proposed to use SV values (which are more commonly used in clinical diagnostics) to estimate a subset of the integrated model parameter values. As a technical solution we proposed the used of Bayesian networks (see Figure 2(c), [35]) to connect immune markers and selected model parameters. This connection was also possible through the use of methodologies and results obtained during Objective 1 such as the ChainRank methodology briefly described elsewhere in the manuscript. However, the generation of accurate linking-by-Bayesian networks is limited by the requirement of a large number of samples. Therefore, to increase accuracy we made use of public available muscle-related data-sets in GEO [6] and included estimates of relations from other sources such as text-mining [36]) into the Bayesian network generation. While we considered that the proposed approach to be technically valid, still we need to increase the sample size to generate useful models, therefore the requirement of follow-up studies. The model can be run in the Synergy-COPD Simulation Environment and the patient specific values can be obtained through a COPD Knowledge Base [2,7].

As a second approach, we integrated transcriptomic data from muscle biopsies and literature-based data into a mathematical discrete model [37]. By this model-driven approach we aimed to determine the processes that lead the abnormal adaptation to training in COPD patients and the role of ROS in this process. Since skeletal muscle mitochondrial dysfunction is a central actor in COPD [38] this approach was based on those genes associated to selected mitochondrial processes from Objective 1's candidate biomarkers obtained in [22]. The modeling was achieved by inferring the activity state of a gene regulatory network (GRN) in six different states: Control group, COPD with normal body mass index (BMI) and COPD with low BMI before and after undergoing 8 weeks of training program [22]. We carried out this task in two parts: 1) GRN reconstructions and 2) Integration of GRN into a discrete model.

As a first step in the GRN reconstruction we curated the list of candidate biomarkers to be included by the re-analysis of the transcriptomic data of the six different states used previously in [22]. For this aim, we used statistical methods such as rank product [39] to determine the gene candidates and Gene Ontology and Human Proteins Atlas databases [40,41] to filter those genes associated with mitochondrial processes in skeletal muscle. Next, to determine gene associations we used IPA software and DroID [42,43]. Finally, In order to correct incomplete or erroneous annotations and identify the direction and the sign of the interactions, we manually curated the GRN reconstruction using a large number of bibliographic data sources.

The GRN reconstruction was then converted into a mathematical discrete model based on the *Thomas formalism *[44] by mechanistically describing the interactions between those mitochondrial-associated genes that were differentially expressed between states. In order to refine the accuracy of our model predictions, we used public available muscle-related data-sets in GEO [6] to impose constraints to our model. We integrated these constraints in the form of inequalities based on probabilistic approaches: if we observed a strong Pearson correlation (*rho*>0.9) between two non-connected genes, their expression values were forced to evolve in the same direction. The rationale is just the opposite in the case of a strong anti-correlation. Then, summing up, we propose a method by which the interaction between genes are determined by performing a tissue and organelle specific GRN reconstruction and the constraints are defined using probabilistic approaches, finally both, the GRN reconstruction and the constraints are integrated into a discrete model in order to unveil the mechanisms governing the adaptation to training in the groups of study.

Together, both probabilistic approaches show a way forward to close the inherent under-determination gap of deterministic, quantitative models by coupling data driven and knowledge driven approaches.

### Objective 3: Clinical application and limitations in Synergy-COPD

The models proposed to address *MusclDys *are still far from the clinical practice. We consider that the methodologies to achieve such goal do exist, but the data publicly available is limited. Several lessons can be learnt:

(1) While the use of mechanistic models is very valid to understand biological systems and diseases, they have serious limitations in the study of complex diseases. Complex diseases may be described as the combination of many factors, and mechanistic models will require too many parameters and consequently too much data to be *yet *clinically effective.

(2) However statistical predictive models (e.g. linear models, Bayesian networks, etc.), not necessarily mechanistically accurate, may provide a valid technical solution. The implementation of such technical solution requires the use of large amount of data to ensure accuracy and statistical validation. For this data to be obtained we consider necessary (1) to strengthen the policies promoting data-sharing (especially in the clinical context) and (2) the generation of large data-sets with proper experimental designs and clinically-driven hypotheses.

(3) Clinically driven research needs to be re-designed to align the different objectives described in Figure 1. We observed that the re-use of data is necessary but complex, and minimal modifications (such as extended questionnaires to patients providing samples) in existing bio-banks may be very useful.

## Understanding COPD co-morbidities through systems medicine

COPD has been associated with several diseases such as lung cancer [45], metabolic syndrome and cardiovascular diseases [46]. However, not all COPD patients share the same diseases or exhibit the same degree of co-morbidity. We hypothesized that the particular co-morbidities in a given COPD patient can be understood from his/her particular set of de-regulated pathways and genes. Identifying genes and pathways that are shared between COPD associated diseases could therefore allow for a more detailed characterization of COPD and its co-morbidities. In the first subsection we briefly describe our method to identify such potential biomarkers (pathways and/or genes). We then introduce our initial predictive models in the second subsection and finally discuss the clinical applications. Some of the data sets supporting this article are available in HuDiNe repository (http://barabasilab.neu.edu/projects/hudine/resource/data/data.html); for the rest we had permission to access and analyze the data.

### Biomarker and Co-morbidity identification

Using 13 million health records from U.S. Medicare [47], we identified 27 disease groups (DG) with significantly elevated risks to co-occur with COPD. These groups included both well-established associations like cardiovascular diseases or lung cancer, but also unexpected ones that could be interesting candidates for more focused follow-up investigations. In order to elucidate possible shared molecular origins between the disease groups and COPD, we considered their respective implicated pathways: for each disease group, we first constructed a comprehensive list of known associated genes from the literature (by pooling several sources of gene-disease associations such as OMIM, NIH Thesaurus and text-mining among others). We then performed a pathway enrichment analysis for each disease group. The results show that there are a number of pathways that are shared between different disease groups, suggesting that the observed co-morbidities are indeed rooted in shared molecular mechanisms. By further inspecting the genes within prevalent pathways we were able to identify a number of genes with the potential to characterize COPD co-morbidity. We are investigating if those markers may predict the level of co-morbidity. This could be of immediate relevance for the clinical practice, as co-morbidity has been associated to lower overall quality of life [48] and increased mortality [47,49]. To date, a number of interesting outcomes of this analysis remain to be validated in further studies. However, with currently available data we considered that the disease groups and co-factors such as age and gender could be used to generate predictive models.

### Predictive models

We selected disease groups that are highly prevalent in COPD patients, such as heart and circulation associated diseases and digestive alterations. The observation that their prevalences vary with age prompted us to develop a first model (Objective 2) aiming to predict the probability for specific co-morbidities in COPD patients over different age strata; in this case we made use of the ranking of co-morbidities from Objective 1 to select those diseases of major interest in the generation of the predictive models. This model may be used as support information for clinicians in the daily practice (e.g. predictive medicine, or comparing observed symptoms with candidate co-morbidities). For a more robust clinical validation, however, follow-up studies in different cohorts will be required; for this reason we consider the comorbidity modeling as part of Objective 2, but closer to the Objective 3 that any other model presented.

### Clinical application and limitations in Synergy-COPD

While co-morbidity is being generally accepted as a relevant clinical factor [47,50-55], co-morbid predictive models are rarely reaching the clinical practice. Our experiences gained throughout the Synergy-COPD project suggest several limitations:

(1) Incompatibilities in the medical nomenclature. While there are several large health registries available to investigate co-morbidities (e.g. Medicare, Swedish Registry and others), there is yet to agree a common diagnostic standard even for simplified administrative coding. In Sweden, for instance, nowadays ICD10 is being used, yet the registry also includes information coded in ICD7 to ICD9. In comparison, Medicare (as used in [47]) is mainly using ICD9 codes. Maps between ICD coding do exist, but they are not accurate, and every new ICD coding system may represent a different conceptual approach. ICD11 will represent a new challenge.

(2) There are many studies investigating specific co-morbidities, not only in COPD but in many other diseases. Yet, two major limitations inhibit the integration of these studies in larger meta-analyses: (1) diseases may be defined differently in each study and in many cases no official coding is followed; (2) the selection of diseases is biased towards well established and expected diseases. These two limitations reflect that most studies are developed to validate specific hypotheses. We believe that broader studies and normalized questionnaires will eventually facilitate meta-analyses and thereby increase the power of co-morbidity studies.

(3) Finally, previous large-scale studies are often limited to one type of data: either -omics data were collected, but no co-morbidity information, or the other way round. We believe that in the future it will be crucial to combine these two approaches.

## Conclusions

Despite the massive amounts of data collected in medical research throughout the last decades, our understanding of complex diseases still remains very limited. The fundamental shortcoming of our knowledge may be illustrated by a popular quote attributed to Ernest Rutherford: *all science is either physics or stamp collecting *[56]. Systems Medicine bears the promise of facilitating the transition from stamps to understanding. Indeed we are convinced that a systems perspective is necessary for the integration of all hitherto largely disconnected facts, thereby ultimately enabling clinically predictive tools. Synergy-COPD presents a large-scale case study of Systems Medicine applied to COPD, recognizing that both *Clinical Research *and *Clinical Decision Systems *require the development of integrative quantitative models. Developing such models is a complex task which we addressed by adhering to a 3-step framework: (1) feature identification, (2) model generation and statistical validation, (3) clinical validation and implementation. We developed and used the framework targeting specifically the characterization of muscle-related systemic effects and co-morbidity as use-cases thus grounding the methodology in real-world applications. In both use-cases we were able to identify candidate biomarkers that may help characterizing COPD heterogeneity, and developed models with the potential to be considered in future Clinical Decision Support Systems (e.g. co-morbidity prevention and prognosis among other objectives). Throughout the project we identified several key factors that are currently limiting the clinical applicability of our approach: the most important ones were data availability, normalization of frameworks (*e.g. *ICD codes in co-morbidity) and the necessity of broader and optimized experimental designs (*e.g. *the inclusion of co-morbidity information in genomic studies).

In conclusion, we consider that the first steps to bridge the gap between basic research and clinical practice are built, however further steps are required to complete the path. To exploit the full potential of our results, future follow-ups are required for statistical and clinical validation, and once validated, predictive models (supported by longitudinal studies) will make a strong case for clinical applications. Further considerations on challenges and future are discussed in [57] on this Supplement.

We are at the juncture of a very exciting era, where Systems Medicine offers the possibility of a real connection between research and clinical applications. While Synergy-COPD may only represent a minor milestone along a long road, we are convinced it is a relevant and instructive case.

## Competing interests

DM is part of Biomax Informatics AG. The rest of authors declare they have no competing interests.

## Authors' contributions

DGC and JM defined an initial draft of the manuscript. DGC, JM, JR, JT, and LB reviewed and defined the final structure. DGC and JM wrote the manuscript. All authors first reviewed their specific sections in detail, then reviewed the full document, in both cases they proposed modifications; finally all authors agreed on the final version.

## Declaration

Publication of this article has been funded by the Synergy-COPD European project (FP7-ICT-270086). The opinions expressed in this manuscript are those of the authors and are not necessarily those of Synergy-COPD project's partners or the European Commission

This article has been published as part of *Journal of Translational Medicine *Volume 12 Supplement 2, 2014: Systems medicine in chronic diseases: COPD as a use case. The full contents of the supplement are available online at http://www.translational-medicine.com/supplements/12/S2.
